# A critical narrative inquiry to understand the impacts of an overdose prevention site on the lives of site users

**DOI:** 10.1186/s12954-020-00458-0

**Published:** 2021-01-06

**Authors:** Abe Oudshoorn, Michelle Sangster Bouck, Melissa McCann, Shamiram Zendo, Helene Berman, Jordan Banninga, Marlene Janzen Le Ber, Zayya Zendo

**Affiliations:** 1 Western University, London, Canada; 2 Middlesex-London Health Unit, London, Canada; 3 Centre for Research On Health Equity and Social Inclusion, London, Canada; 4 School of Leadership and Social Change, Brescia University College, London, Canada

**Keywords:** Harm reduction, Narrative inquiry, Overdose prevention site, Supervised consumption site, Photo, Relationships

## Abstract

**Background:**

Globally, communities are struggling to gain support for harm reduction strategies being implemented to address the impacts of substance use. A key part of this discussion is understanding and engaging with people who use drugs to help shape community harm reduction strategies. This study focused on how an overdose prevention site has influenced the lives of people who use drugs.

**Methods:**

A critical narrative method was utilized, centred on photo-narratives. Twenty-seven individuals accessing an overdose prevention site were recruited to participate in preliminary interviews. Sixteen participants subsequently took photographs to describe the impact of the site and participated in a second round of interviews. Through independent coding and several rounds of team analysis, four themes were proposed to constitute a core narrative encompassing the diverse experiences of participants.

**Results:**

A key message shared by participants was the sense that their lives have improved since accessing the site. The core narrative proposed is presented in a series of four themes or “chapters”: Enduring, Accessing Safety, Connecting and Belonging, and Transforming. The chapters follow a series of transitions, revealing a journey that participants presented through their own eyes: one of moving from utter despair to hope, opportunity, and inclusion. Where at the outset participants were simply trying to survive the challenges of chaotic substance use, through the relationships and services provided at the site they moved towards small or large life transformations.

**Conclusions:**

This study contributes to an enhanced understanding of how caring relationships with staff at the overdose prevention site impacted site users’ sense of self. We propose that caring relationships are an intervention in and of themselves, and that these relationships contribute to transformation that extends far beyond the public health outcomes of disease reduction. The caring relationships at the site can be a starting point for significant social changes. However, the micro-environment that existed within the site needs to extend beyond its walls for true transformative change to take place. The marginalization and stigmatization that people who use drugs experience outside these sites remains a constant barrier to achieving stability in their lives.

## Background

Communities globally are experiencing the devastating impact of the drug overdose crisis. For the first time in over 4 decades in Canada, life expectancy among males and females did not increase from 2016 to 2017 (Statistics Canada, 2019). One of the major contributing factors to the drug overdose crisis is opioid use. In terms of morbidity, the rates of emergency department visits and opioid-related hospitalizations have been increasing [26] representing a large burden on health care systems. In response to this intensifying crisis, multiple community and government agencies have joined in local efforts to save lives and address harms associated with opioid use.

Overdose prevention sites or supervised consumption facilities are a public health, harm reduction strategy to address the health needs of persons who use drugs (PWUD). These are sites where people can consume their own substances in a safe environment within the presence of harm reduction staff, medical supervision, and peer workers. While research has focused on the health outcomes related to overdose prevention sites, these do not necessarily capture the full human impact of these services. Therefore, the purpose of this study is to add depth of understanding through narrative analysis of the experiences of people who use this type of facility and how that use has influenced them. This was done through the co-creation of knowledge by collecting the participants’ stories using semi-structured interviews and photographs taken by them. Our specific research question was “How has this overdose prevention site changed the lives of those who have accessed the site?” We were interested in hearing about all types of changes including behaviours, relationships, interactions with others, feelings, and perceptions of self and others. The findings from this study deepen our understanding of the experiences of individuals who use such sites and the role of these sites in the community.

The evidence base regarding overdose prevention sites and supervised consumption facilities is developing. The focus of research is often on reducing substance-related fatalities and might include cost–benefit analyses [9]. However, beyond these surface-level outcomes, there are clear health equity impacts of such sites. Research has shown that the primary users of supervised injection services are those who are most marginalized [25]. A recent systematic review of supervised consumption facilities suggests that supervised consumption facilities are effective at meeting their public health objectives of mitigating overdose-related mortality; reducing substance-related risk behaviours such as syringe sharing, syringe reuse, injecting outdoors, and rushed injections; and facilitating uptake of addiction treatment and other health care services [16]. Furthermore, the review suggests improvements in public order outcomes such as reduced public injecting, reduced publicly discarded syringes, and reduced injection-related litter without increasing substance-related crime [16].

The meaning of the sites to PWUD is more than just a regular health service. The concept of overdose prevention sites as a safe place for PWUD is well-established in the research literature. A review of forty-seven qualitative studies identified the key benefits of injection sites as perceived by PWUD are “safe place”, “safety”, and “education” [20]. Furthermore, a meta-analysis of twenty-one qualitative studies found that PWUD perceive these sites as safe, regulated spaces they could comfortably occupy, as opposed to other public or private spaces [22]. These sites were free from violence and real or perceived stigma, which created a safe micro-environment for site users. While being primarily set up to influence health outcomes, from the perspective of site users, they were first and foremost safe environments [22]. This begins to demonstrate the potential impacts of overdose prevention sites beyond medical outcomes. These safe environments help PWUD to feel comfortable engaging with staff about their needs. It has been suggested that this supportive environment comes about because the sites have “disrupted stigmatization processes and improved trust in programme staff [22, p. 156]”. This fostered trust facilitates acceptance of other supports such as food, shelter, and broader medical and social supports [14, 22]. Accessing overdose prevention sites has had a notable positive impact on the broader determinants of health, such as increasing access to housing for those experiencing homelessness [17].

Despite the effectiveness of overdose prevention sites in addressing public health outcomes, improving public order, and the acceptance of the sites by the PWUD, the implementation of overdose prevention sites remains controversial. Political climate, community perceptions, and the stigma of substance use, have significantly impacted the implementation process [1]. There is potential for transforming stigmatized public perspectives if we better understand the transformational impacts of overdose prevention sites beyond just health improvements. To do so requires in-depth, qualitative understanding of the experiences of those who use such sites.

## Methods

Critical narrative inquiry, an approach that generates insights by examining the interplay of narratives, discourses, and power dynamics, was the over-arching methodological approach. Studies [3, 4, 28] have shown the merits of this approach as a means to explore the life stories of participants. Within this study, critical narrative inquiry allowed us to more deeply understand how a local overdose prevention site had influenced the lives of site users, their relationships, interactions with others, and perceptions of self. Critical narrative inquiry allowed for a space for participants to construct their life story and their realities in ways that made sense for them [21]. In terms of the particular narrative approach, we chose to use a photo-narrative method. A variety of photo-based approaches are currently used in research, including photo-narrative, participatory photo-interview, and photovoice [7, 13, 30, 32, 33]. While there are variations within each of these methods, common to all is an invitation to the participant to address research questions by taking photographs and discussing them with the research team. In a sense, photographs are an “ice-breaker”, a medium that creates a comfortable and safe space for discussion [6]. In our design, we opted for the term “photo-narrative” to emphasize the participants’ independent capacity to take their own photographs and describe them during an interview [11]. This process meant that the participants went beyond providing simple descriptions of the photographs but rather constructed their personal journey or “plot” [29] through explaining the photos.

The overdose prevention site of study operates legally in Canada and is run by a multi-service organization focused on the needs of those living with or at risk of HIV/AIDS or Hepatitis C. Site staff work in the drug consumption room to support site users and provide education about substance use practices, as well as potential health concerns from injection drug use. The site is supported by several community partner agencies that provide wrap-around health and social services on a rotational basis at the site.

Potential participants were first informed of the study by site staff who conducted a preliminary screening of potential participants in order to determine eligibility for participation.

If a potential participant indicated interest, the site staff accompanied them to an interview room where they were introduced to the researchers. A total of four researchers collected the data. The researchers worked in pairs when conducting interviews with the participants during designated data collection times at the site. Site staff also supported the researchers in achieving maximum sample variation using a purposeful sampling method. This was done in order to ensure a diverse study sample was obtained, according to age, gender, race, ethnicity, length of site use, duration of substance use, substance being used, housing status, source of income, and chronic health challenge.

Participants took part in a first interview during which they were asked to describe their day-to-day life, and what the site meant to them. The questions were developed to get participants to think about how the site has changed their lives, behaviours, relationships, and perceptions of self and others. Participants were then given disposable or digital cameras and photo-taking instructions to ensure photography etiquette and encourage the use of photographs that do not identify people. An inspirational board was also used to assist participants to think about the types of photographs to take and provide some direction to participants. All interviews were audio-recorded. After each interview, both researchers present discussed the key findings from the interview and documented their impressions on a debriefing form. Two participants conducted their interviews together given their intimate relationship with one another, and another participant asked to have a friend present for support during the first interview.

Participants were then given up to 1 week to take photographs, and these were developed upon return of the cameras. For ten of our participants, a Peer Support Worker accompanied them as they took photographs on a digital camera. This was a change to the data collection protocol introduced to increase participant completion of the second interview by reducing the time for photograph development and the delay between first and second interviews. The purpose of this second interview was to discuss the developed photographs in a semi-structured interview. In most cases, participants met with at least one of the two researchers who conducted the first interview. At this stage of the data collection, participants were asked to select their five most meaningful photographs that best captured how the site had changed their lives. Participants were asked to provide a description of each of the photographs and to share why and how they decided to take that particular image. Researchers asked questions to explore more deeply the significance of these photographs. Participants received a $25 honorarium for each interview in which they participated.

Interview audio recordings were professionally transcribed verbatim for analysis by the research team. Field notes and debriefing forms completed during the interviews were also reviewed as part of the data analysis process. The analysis was conducted by the research team members in a multi-phased process involving individual and group analysis. The overall goal of analysis was to generate a core narrative [28] supported by key themes. A core narrative is somewhat similar to grounded theory in that it aims to produce explanations regarding the accounts and patterns of behaviour that are relevant and problematic to those involved. The intent is to generate explanations of experiences around core categories [12]. In this study, the core categories emerged through thematic analysis, where the focus was to explicate the meaning of the text, emphasizing what was being said, rather than how it was being said. In essence, the analysis strategy employed included both a narrative analysis and a thematic analysis.

For member-checking, the researcher team presented the proposed core narrative with themes to community stakeholders, research participants, and site staff throughout an afternoon. Feedback was overwhelmingly positive regarding both the structure and the content of the proposed findings, and this process of engagement helped us to refine the terminology of the themes. The final step of analysis occurred through the collaborative writing of the final report.

In terms of the sample, 27 initial interviews were conducted, followed by 15 follow-up interviews (two participants were interviewed together for a total of 16 participants) where participants’ photographs were discussed. Of the 27 participants, 59% (16) were male, and 41% (11) were female. Half (14) of the participants were homeless, and one-third (9) identified as indigenous. A detailed intersectional analysis related to indigeneity, gender, and age is being conducted for a separate manuscript. Participants self-identified as homeless or housed and if they asked for clarification, the Canadian definition of homelessness was used, including unsheltered, emergency sheltered, or provisionally accommodated. Almost all participants had used substances for more than 10 years, with opioids being the most commonly used substance. Characteristics of the 16 participants with a complete data set (first and second interviews along with photographs) were very similar to the initial 27 participants interviewed. However, two characteristics were slightly different. A greater percentage of participants with a complete data set were homeless (69% vs 52%) and 88% had used substances for more than 10 years, compared to 75% of the full sample. Detailed participant characteristics are presented in Table 1.

**Table 1:** Participant characteristics

Variable	Characteristics	All participants (*n* = 27)	Participants who completed both interviews (*n* = 16)
Gender	Female	41% (11)	44% (7)
	Male	59% (16)	56% (9)
Age	20–29 years	30% (8)	31% (5)
	30–39 years	30% (8)	31% (5)
	40–49 years	33% (9)	31% (5)
	> 50 years	7% (2)	6% (1)
Ethnicity	Indigenous	33% (9)	31% (5)
	Non-indigenous	67% (18)	69% (11)
Housing status	Homeless	52% (14)	69% (11)
	Housed	48% (13)	31% (5)
Income source	Social assistance^a^	100% (27)	100% (16)
	Ontario works	60% (16)	63% (10)
	Ontario disability support program	33% (9)	31% (5)
	Other	7% (2)	6% (1)
Length of substance use	< 5 years	7% (2)	–
	5–10 years	19% (5)	13% (2)
	11–20 years	41% (11)	50% (8)
	> 20 years	33% (9)	38% (6)
Substance used	Opioids	63% (17)	56% (9)
	Crystal meth	19% (5)	25% (4)
	Ritalin	4% (1)	6% (1)
	Combination	15% (4)	13% (2)

^a^In addition to social assistance, some participants included other sources such as busking, hustling, panhandling, returning empties, selling art, and stealing

## Results

A critical component of narrative analysis is the articulation of a core narrative, the story that combines and underpins all the participant stories [27]. The core narrative we are proposing is a chronological process. It is important to note that while this narrative does not represent the precise experiences of all participants, and that some participant stories were far less linear, the arc of this story is what we have perceived the participants were relaying to us. Each journey shared with us was unique and many journeys included moves back and forth along this arc. An experience of belonging or transformation might be as brief as the length of a visit to the site, a few days or longer. We also acknowledge the diverse social locations inhabited by our participants, such as gender, race, ethnicity, age, and ability. A diverse sample was deliberately included to ensure we made space to understand the perspectives across varying identities. These intersecting identities influenced both experiences leading into substance use and the lived journey of accessing site services. For example, it is recognized that women face unique challenges that have led them to substance use and continue to shape their experiences (e.g. sexual abuse).

**Fig. 1:**
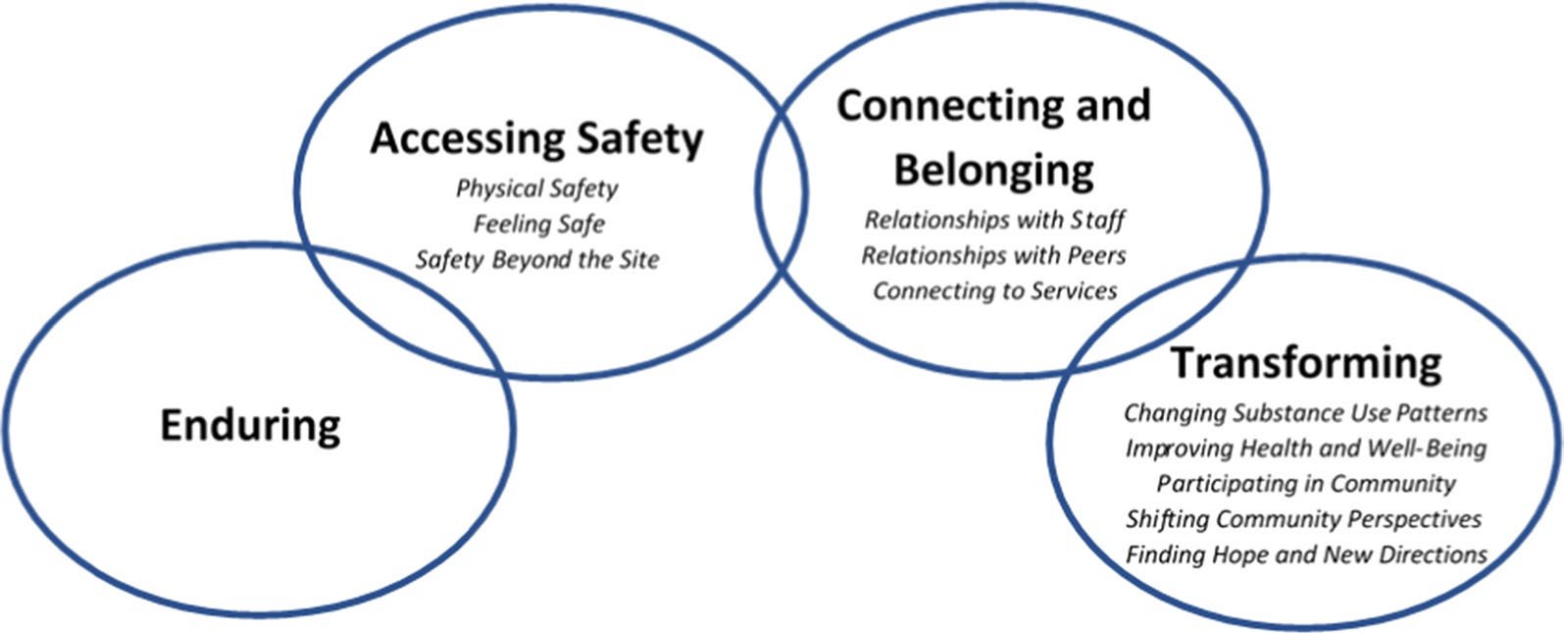
Visual depiction of core narrative arc and identified sub-themes

The clear message shared with us by participants was the sense of their lives having improved since or while accessing the site, often in unexpected ways. We have chosen to present this core narrative in a series of four themes or chapters: *Enduring, Accessing Safety, Connecting and Belonging*, and *Transforming*. We were drawn to the analogy of chapters of a book by the tangible differences in experiences across a series of key transitions in the lives of participants. The chapters follow these transitions, unfolding a journey that participants presented to us through their eyes; one of moving from utter despair to hope, opportunity, and inclusion. Ultimately, the site and staff had transformative and unexpected impacts on the lives of the site users they served (Fig. 1).

### Chapter 1: Enduring

*Enduring* represents the experiences of participants prior to accessing the site and to some extent, their experiences that continue when not at the site. It reflects the sorrows, traumas, despair, and hopelessness experienced in the midst of chaotic substance use. However, as the participants eloquently shared with us, their lives were also characterized by strengths. In the midst of this suffering, they found creative ways to survive.

A common idea that underscored the experiences of enduring was the lack of potential or possibility. Addiction itself was perceived as a near-insurmountable barrier to living life any other way. Substance use was a dead-end, a downward spiral, a weight or chain:
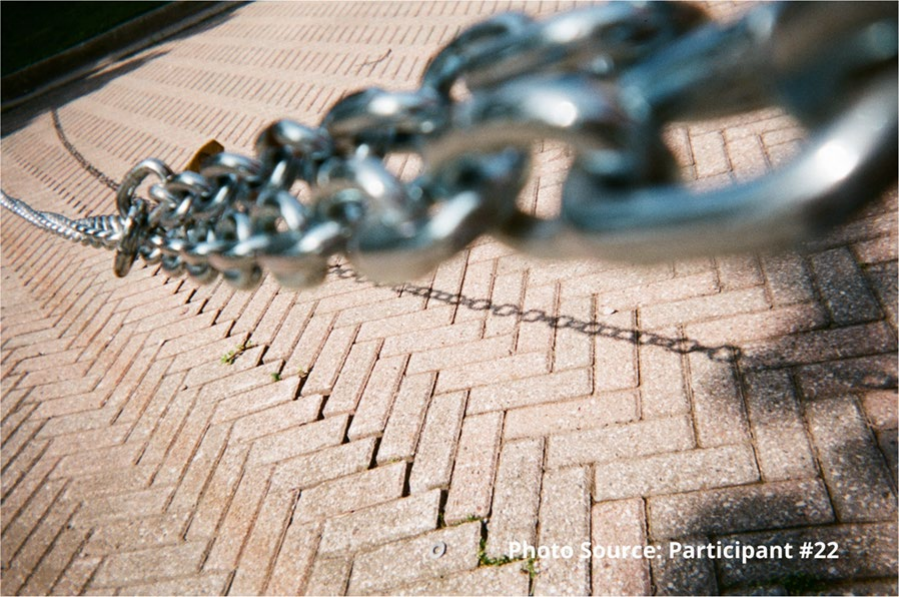

“I feel like I’m always in chains…. The addiction is like you’re in a ball and chain every day of your life.” – Participant #22

This participant also shared an image of a staircase to represent that sense of going downhill:
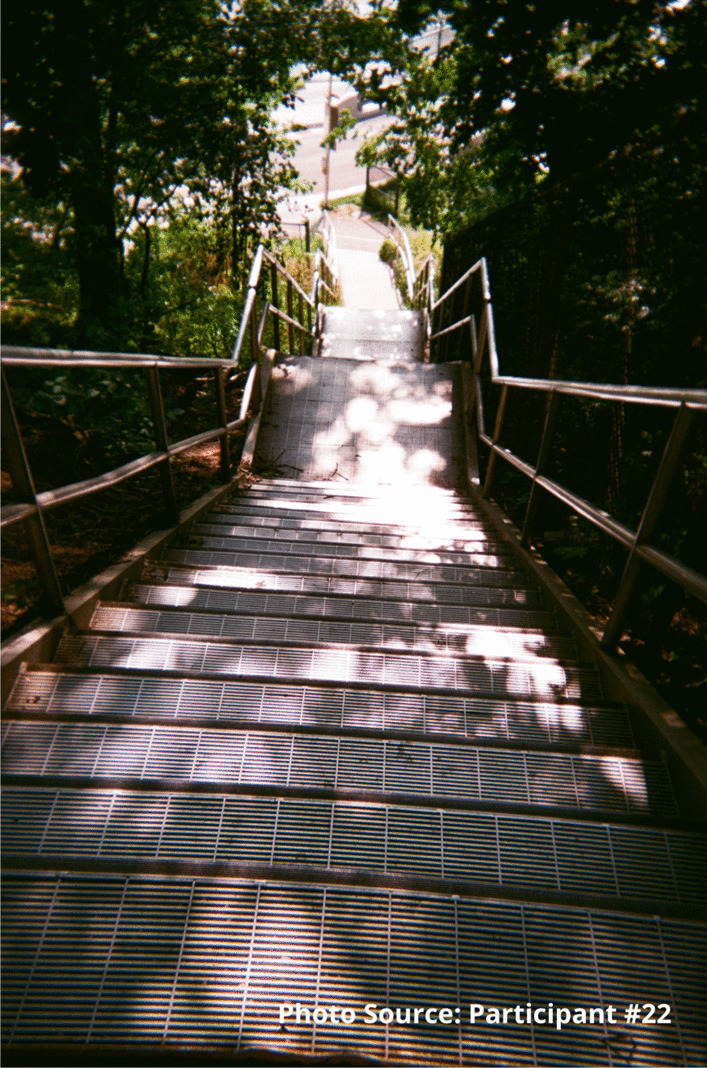

“I took the picture of the stairs going down because to me it was like I was going down you know what I mean, like I was taking a trip down somewhere not good. Like for example I could use this as I was going into the woods to find somewhere to shoot up. I was going by myself somewhere unsafe right, like this site, like it’s a wooded area you know what I mean, and I didn’t have that much concern for myself at that time because no one was around to tell me that I was worth having concern for myself at that time. So, I took a picture of this going down, right.”

Enduring encompassed significant risks to safety. Some participants shared stories about the risks associated with acquiring funds to purchase substances. It was noted that this was a gendered experience. While all activities to acquire funds involved some risk, women faced particular risks to their safety and well-being at street level, including panhandling and human trafficking. When asked if there were any additional income sources other than social assistance, a few women were uncomfortable providing an explanation regarding their income sources and one female participant became emotional and unable to answer.

Participants also spoke about the harms associated with substance use outside of an overdose prevention site. These included being rushed to inject, reusing gear, using discarded gear, using unsterilized water, injecting alone, being arrested, and being beaten or robbed:

“I more or less had to stay up, do a lot of crystal just so my shit wouldn’t get robbed, and finally at a point where I would crash it never failed, I would end up getting robbed.” – Participant #05

Risks that participants endured included interactions with the justice system. Participants spoke of being in constant fear of negative interactions with police. These occurrences were unintentional but precipitated by having nowhere to use substances other than in public spaces or trespassing on private property. A participant shared an image of spaces they found for using substances prior to the opening of the site:
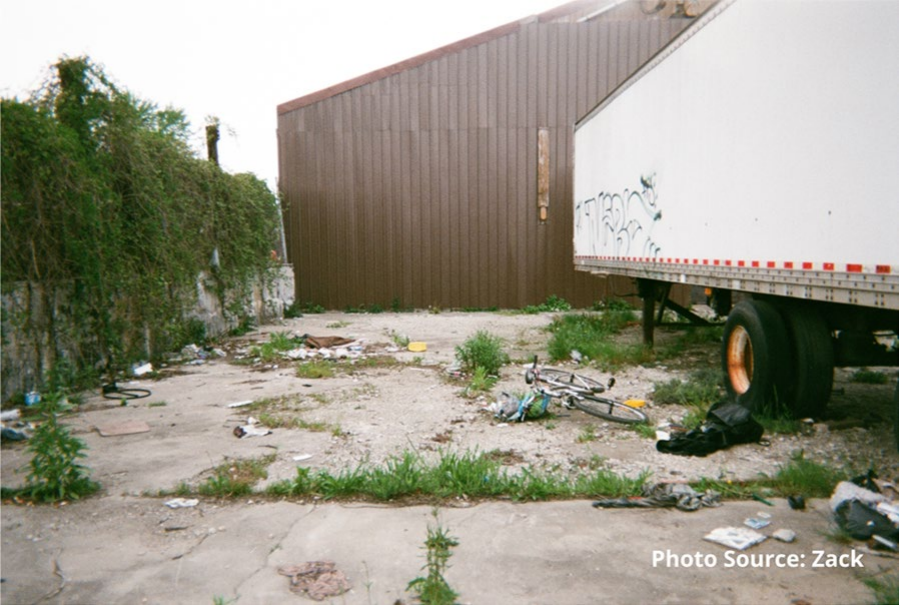

"People are rushed here because you know, cops could drive by at any moment. Like I said a mother and children could walk by any moment and you go oh shit, let’s go and they just leave everything and take off." - Zack

Enduring also involved the trauma of seeing others, sometimes friends or peers, harmed by substance use. Ultimately, enduring the traumas of substance use left participants feeling that they had nowhere to go:
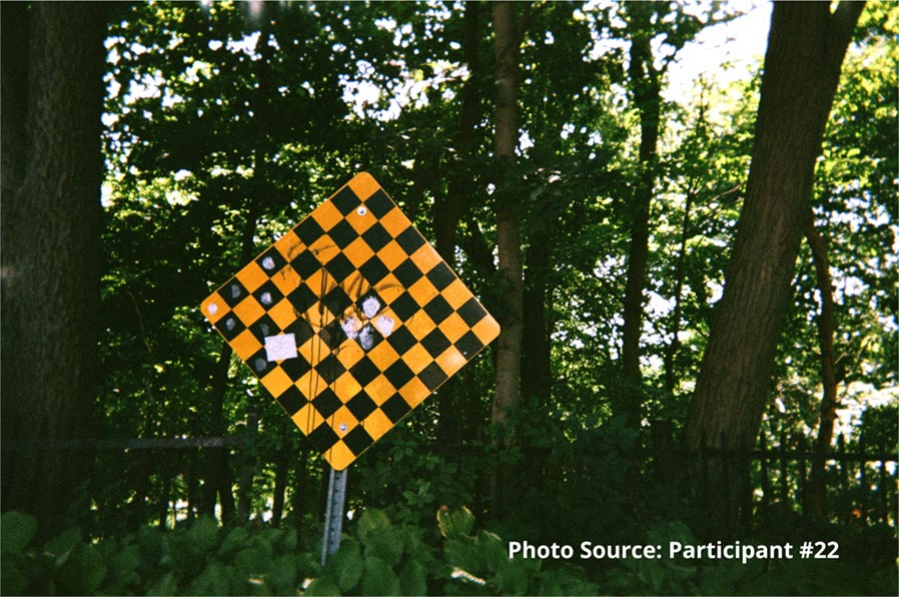

“And it’s a dead end, right. These are the same street. Like I felt like my life was at a dead end. I wasn’t going to, they weren’t going to bust open the woods and make another road to continue on to a good road. My life had stopped and that’s the way I felt before.... Again, I will say again, every time I walk through this door this place saves my life, whether I know it, realize it and need to know it or not, they do.” - Participant #22

While participants highlighted that they survived the multiple challenges associated with substance use prior to the opening of the site, doing so was extremely difficult and was typically accompanied by significant isolation, oppression, and ultimately, risk of dying. Before coming to the site, the only relationships many participants had were, from their perspective, precarious relationships with other peers. Many had experienced overdoses or had witnessed friends die due to overdose or other health complications linked to substance use. For some participants, these times were too difficult to talk about; those who did talk about this time of enduring illustrated how, in deep despair related to substance use, they stopped caring for themselves or others. Instead, they turned to substances in ways they knew put them at risk of overdose or heightened susceptibility to infectious diseases. Without a hope for the future there was no reason to care for oneself or others.

### Chapter 2: Accessing safety

From a public health perspective, the key priority of an overdose prevention site is to reduce the risks of infectious diseases and minimize the risks of death from drug overdose. These ideas are operationalized in practice through the creation of safe, clean, and secure physical spaces for people to use substances. The findings of this study indicated that, as expected, participants accessed the site for safety related to cleanliness and to have a safe physical space where they could use substances under supervised conditions. However, in addition to physical safety, participants described feeling safer at the site. Finally, safer substance use practices extended beyond the doors of the site.

When we think of a journey towards safer substance use, it is common to focus on physical components of safety. Participants spoke frequently to physical safety related to services provided through the site, including decreased risk of disease transmission and overdose. Additionally, there is another dimension of safety associated with harm reduction that is equally, if not more important, namely a general feeling of being safe. Participants described how the site served as a safe place where they could be free of stigma and judgment, and where the stresses of the chaos of living with substance use were quieted, at least momentarily. This is noted as participants talked about more than *being* safe but also *feeling* safe. This safety means that folks are relaxed and more likely to engage in practices that also support physical safety:

“You come in and you feel relaxed. Like I said before its different. It’s hard to explain, but it’s different. You want to come here. Before you would just come here get needles and go. Now they test you for HIV here, they have everything. Like these things which you’re doing right here, stuff like that, awareness and trying to figure out stuff and like it helps the community. Before it was just come here, get my needles and go.” – Participant #20

This is again connected with avoiding unwanted interactions while using in public spaces:

"It’s a really comfortable place, just relaxing place to go and you don’t have to worry about you know, I feel weird saying it, you don’t have to worry about cops, I don’t worry about them but yea, you don’t have to worry about cops rolling up on you and making you uncomfortable and you don’t have to worry about people wondering what the hell you’re doing in a parking lot for no reason" – Participant #26

Participants felt the site staff worked diligently to ensure that the site users derived a sense of safety and comfort. As participants felt more relaxed about their substance use, and not worrying about where to use, finding equipment, or getting arrested, they were learning how to manage psychological stressors. For Shone, the site was viewed as a safety net; staff were there to talk to about his addiction without being judgmental.
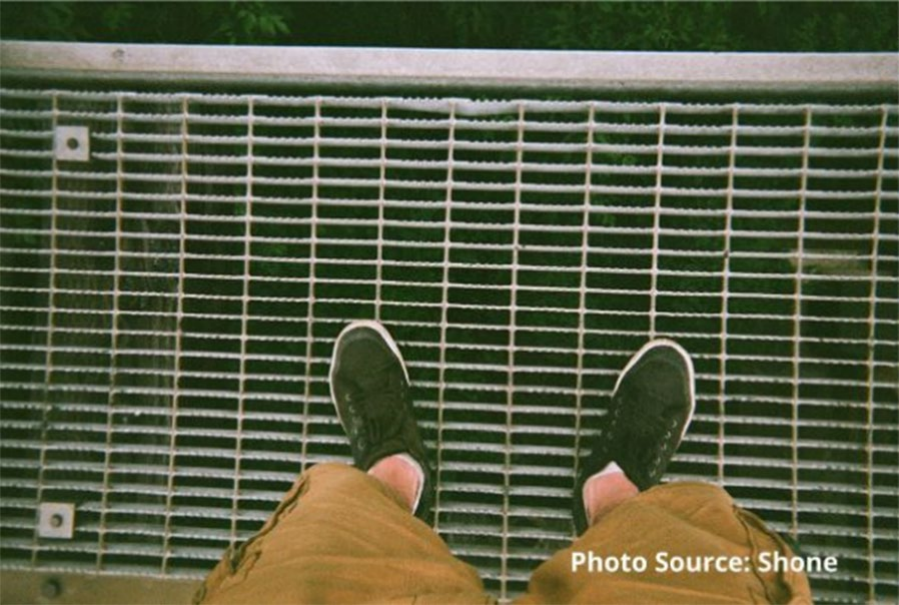

“Because this place is, believe it or not, you know when I got clean, this was my safety net.” - Shone

Shone explained the value of the non-judgmental approach of staff. According to Shone, this approach served to counter the negative internal belief patterns that have developed from years of societal stigma and discrimination experienced as a person who uses substances:

“It was more so all from the social stigma of it, right. You know what I mean, like that kind of, like you internalize it and it becomes just like, I hate saying this but the term junkie, you know what I mean, and you start accepting that term and just living by that because you’re limited by that social stigma. Like whereas with a healthy environment of understanding people, you’re human again.” - Shone

While much of the discussion with participants focused on the safety within the doors of the site, participants also highlighted how safer substance use practices extended beyond the site. A participant spoke to a picture of streetlights and connected this to safety:
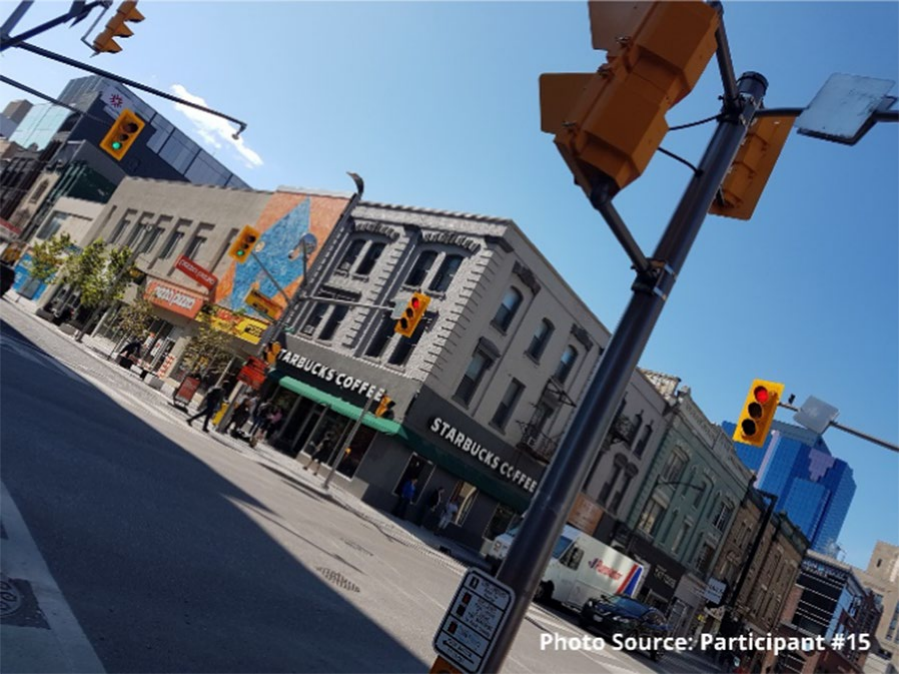

“Streetlights are supposed to be to keep you safe so you don’t get hit by cars right, and I was like how [site] is like a safer place, to me it feels like a safer place to be.... They have like the site here, they have, even if you’re not using the site they have the option that you can pick up and take with you so that way when you are away from the site you can still be safe and at least have like, at least know you have something to try and help you if something does go wrong.” – Participant #15

The lifesaving nature of this extension of support was not just related to picking up new gear but was also related to how site users were being trained on and supplied with Naloxone. This meant that site users were becoming active in responding to overdoses throughout the community. Unprompted, participants reported personally reversing a number of overdoses:

“I’ve saved probably ten people with the Naloxone that I’ve got here, yea, I’ve seen a lot of overdoses, yea.” - Emma

The value of this new-found ability to save each other’s lives was noted in a context where the site is not open overnight, a time of high risk for overdose. Some participants believed that the site has helped to positively shift community morale and has enhanced the level of comradery among the peer community:

“So the morale, the crowd, it just changed when the site came. I found everybody was more welcoming amongst each other... The outside site too, yea, I found it, like I mean if you’re sitting inside the site and you start talking, shoot the breeze or you’re both using or whatnot, you know, you have more of a chance of talking when you’re out and saying hi when you pass versus just passing. So I found, at least I thought it gave us more comradery than I haven’t seen before. So that was cool and that was really good.” – Participant #22

### Chapter 3: Connecting and belonging

Connecting and belonging were central to the experiences shared by participants at the site. This was the next part of the unexpected journey that participants took from the hopelessness of unsupported substance use, to reaching out for safer use, to connecting and belonging. Herein, the key theme is relationships. Relationships were the foundation from which participants journeyed from seeking safety to finding connections and belonging. New relationships were established in ways that had previously eluded our participants. These relationships became an incentive for participants to come to the site whether or not they were, at that moment, in need of harm reduction support:

"But I like to come to this place to socialize and to talk about you know, like I like the peers around me and I like the people that work here. They help me out with more than just supplies or a safe place to use the substances. They actually, I consult with them on things I have to do and you know ask for favours or reminders and even if I need a drink or a bus ticket I’ll come here " - Zack

Participants described how the staff developed genuine relationships that were in contrast to the types of relationships they experienced outside the site. The relationships developed with staff helped to fill a void in participants’ lives by providing a sense of community. These relationships with staff created a place where they felt like they belonged and were accepted unconditionally:

“Well with the site, like I feel it’s more a sense of community rather than make, I speak for myself, but I think a lot of people downtown are searching for community and acceptance and to fit in, to have somebody, and when you do that out there its not real. They say they care about you, but they don’t. They care about your drugs and your money. But here they care about your well-being, who you are. I have a good relationship with a lot of staff here. They know a lot about me and they care.” – Participant #23

Many participants stated this was the first time they had formed a trusting, meaningful connection to a health or social service provider. Staff were consistently referred to as friends and family. While many grappled with the idea of calling staff “friends” or “family” as they understood professional boundaries, these were still the terms that they felt best described the value of these relationships in their lives.

“These people [staff] treat us with respect and we don’t get that anywhere else. Most of us not even from our families you know. This is our families, these are our friends. Like for me I don’t have any friends. These are my friends so to speak. If I have a problem I come here.” – Participant #22

One participant highlighted the importance of staff relationships in leading to changes in his life related to substance use. He represented this idea in a photograph of a bridge. As he explained, the bridge symbolizes the hurdle of getting through his addiction and the support he receives from the staff. The staff are viewed as his bridge to get through his addiction.
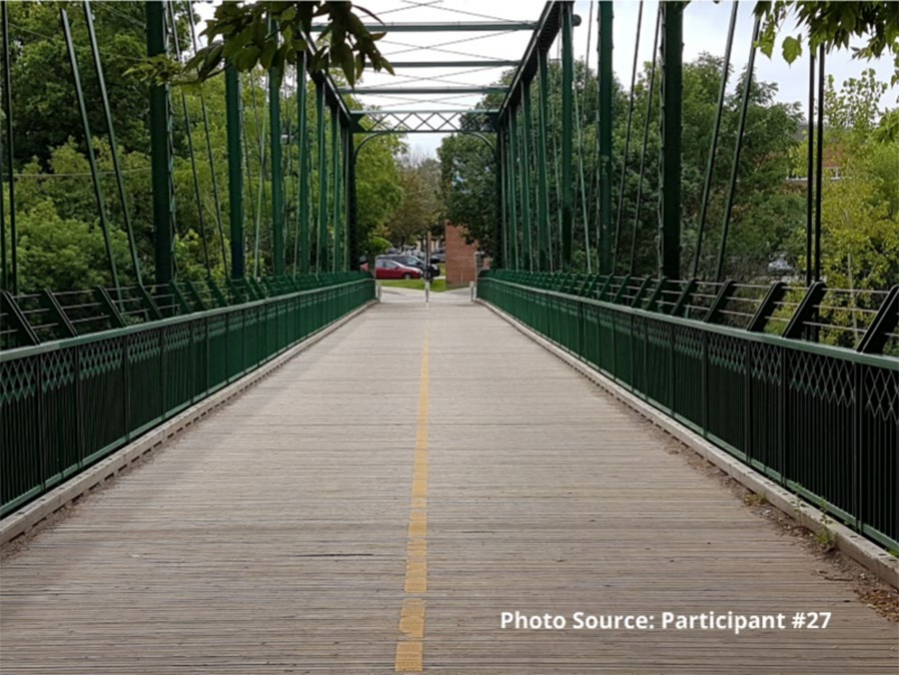


“Just the hurdle of getting through what I’ve been going through and again like how the support of the staff and like [staff member 1] and [staff member 2] and like everybody in their own way has helped me along... They want the best for you. They want to make things easier for you and yea, they’re like that lifeline, that lifeline that you may not have any place but here.” - Participant #27

Several study participants spoke about the sense of loneliness in their everyday lives; however, these same participants described how much they value being able to talk with the staff to help reduce some of the feelings of loneliness. Many participants described the site as a place to come, not only to use substances in a safe environment, but also because it offers opportunities for connection with others when they need a trusted person with whom to talk:

It’s just good to have a place to come to, like if you’re just feeling alone and you just want to talk to somebody, you know. Because without this place you don’t get that.” – Participant 27

As articulated in *Enduring*, the relationships among the peer community were characterized by some participants as precarious and mistrusting. Some described the fear of having their belongings stolen by peers; others reported that they had experienced physical acts of violence. Yet, despite their fears and negative experiences, many also spoke about a strong sense of community that exists among their peer community because of their common experiences of homelessness, addiction, and stigmatization. In particular, some participants asserted that their relationships with their peers have become more positive since the site opened. It appears that the site provided a space where a different type of relationship could be formed than was previously possible.

For some participants, the relationships among peers were strengthened, not only within the site, but also outside, in the broader community. One participant described how the site provided a place where he had established stronger connections and community with his peers. The photograph was taken to illustrate how the site has not only brought peers together, but how these relationships extended outside of the site as well. He has established friendships with many peers and feels like he can see positive change in people’s behaviours, including safer substance use practices:
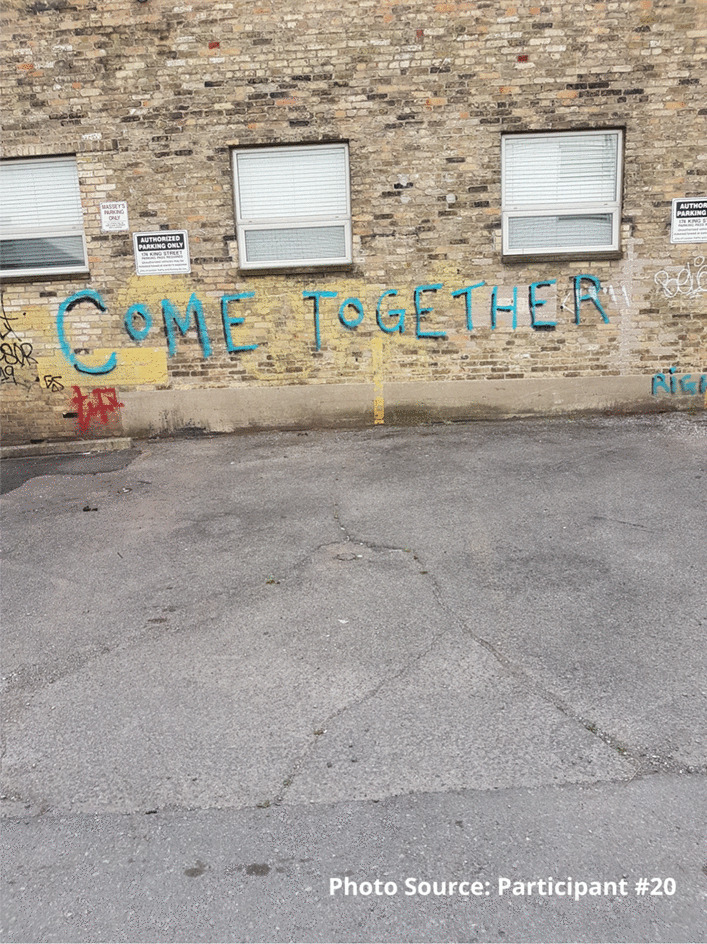

“It brings us all together, we all come together outside, we all become friends you know.” - Participant #20

“We talk inside because we get to know each other right because we’re all talking between each other.... Ever since this place opened a lot of people do things differently.” - Participant #20.

Another participant drew a connection between the value she feels in the development of both staff and peer relationships. She described the strong relationship that she has with her peers noting that these relationships are possible because of her connection with staff.

The development of the supportive and trusting relationships with staff at the site has encouraged many participants to follow the recommendations from staff and seek services for supports that they may not have felt comfortable accessing previously for various reasons. As this participant suggests, some of their interactions with other health and social services are very different.

“Well it just makes me know that people care. And that’s a big thing when you’re a user. You get treated like shit at the hospitals even where you know, and its nice to, and sometimes I just like to come in here just to feel the closeness of everyone you know, because they do care and its nice just to be around people that do care about you, so its great. - Mike

The wrap-around service model is central to the service delivery at the site and helps site users to facilitate relationships being built with not only harm reduction staff, but also medical staff, and staff who rotate in the aftercare room (e.g. housing support staff, addiction support staff, etc.).

“They help you find housing and stuff, and like they’re just really nice, to have someone that, like I said, that’s not judgmental, they actually listen to what you have to say, and they support you. They give you like hope which is something I never had before in my life.” - Participant #15

Through their words and pictures, participants in this study showed how fundamental relationships are to the service delivery at the site. The relationships with staff have significantly influenced their day-to-day lives. Through these relationships, site users have found connection and belonging with staff and with their peers. They have also been connected to services that in the past they were either fearful of or did not know they existed. This connection and belonging is the next step on a journey towards transforming.

### Chapter 4: Transforming

Participants came to the site for safety and found physical safety, but also discovered a deep sense of belonging and even transformations. These transformations were evident in many respects, but particularly prominent were changes in substance use practices, health and well-being, community participation, and hope for the future. The key theme herein is *Transforming* and includes processes of *changing substance use, improving health and well-being, creating civic engagement, shifting community perspectives,* and *finding hope and new directions*. The experiences of *Safety* and *Relationships* are the foundations that make transforming possible. How each participant experienced change in their day-to-day life because of coming to the site was unique to them. For many participants, their experiences at the site provided opportunities for growth, hope, and transformation of themselves and their community.

As articulated in *Accessing Safety*, participants came to the site and adopted safer substance use practices. As participants adopted these safer practices, it changed their substance use behaviours both within and outside of the site:

“Using clean equipment, like using clean needles. I put a clean cooker and clean water. It teaches me to always use clean equipment so I don’t catch HIV and I don’t catch HEP C.... I don’t have HIV and I don’t have HEP C but it could happen, like I don’t share nothing, no cookers anymore. I used to do like people’s washes but now I don’t because I know you can still get diseases through it and they’re the ones that taught me that.” – Participant #20

Some participants also started talking about changes to when, how much and what substances they used. It is vital to consider that as a result of this support, many participants stated that they were less likely to use substances to manage their psychological stressors. That is, for some participants, simply having positive relationships at the site was directly linked to reduced substance use. Feeling safe through respectful relationships emerged as one pathway for some participants to journey through recovery. This function is quite separate from more formal treatment service connections offered through the site.

“No, it’s definitely had an impact. I think with the resources and the way that these, the way that they make you feel and just, I can walk out of here and not use for hours because they make me feel like I’m a person again, right. Like that’s awesome. Because otherwise when I’m not here, I’ll use every hour if I could because something will make me feel down. When something makes me feel down I want to use, I want to use. And I come here and I’ll chat with these guys and laugh, or like be able to vent on something bad that happened; I’m okay for a couple hours. And it really has changed that.” - Participant #22.

Other participants spoke of the staff support they have had in accessing services. For one participant, accessing housing and receiving drug replacement therapy were critical factors that helped reduce the chaos in his day-to-day life:
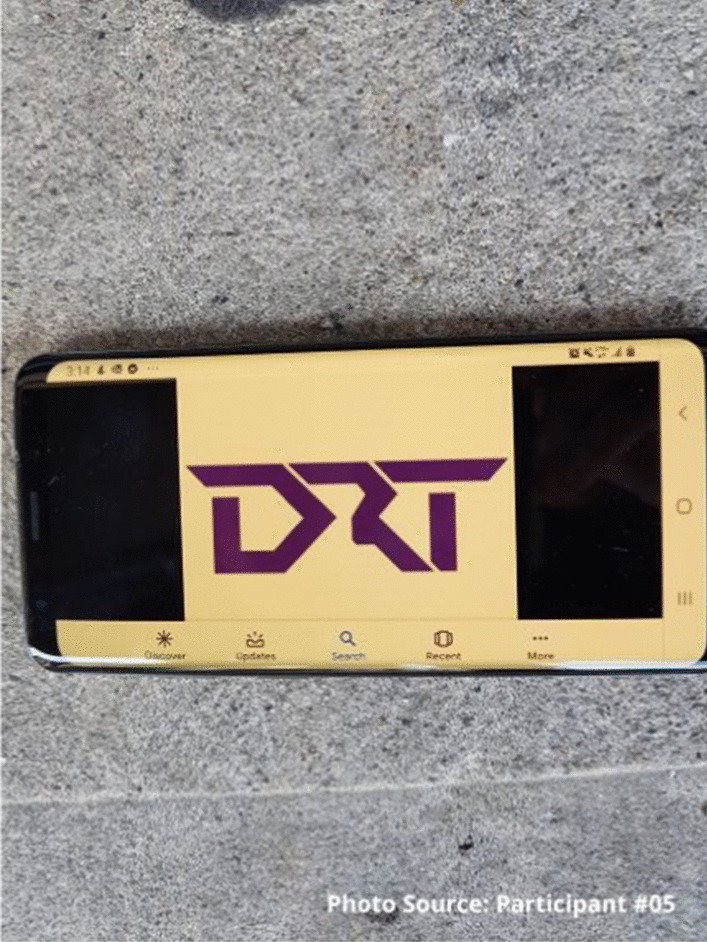

“Just not chasing the drug, you’re not looking for the drug every day and spending most of your time finding it and getting it.... I’m just slowly getting organized. I got my place, I’m going to try and get stable, like a job, I want to retain a job. This has helped a lot.” – Participant #5

The relationships and supports that participants obtained from site staff helped them make the changes to their substance use patterns that they desired. Stable housing and/or access to drug replacement therapy/safe supply also appeared to be important factors for some participants as they attempted to transform their substance use.

In addition to the direct changes to substance use behaviours, many of the day-to-day changes identified by participants positively affected their well-being. A participant explained that the safety and comfort of the site allowed people to let go of the constant negativity and stigma they perceived within the community. In the site, people experienced a space where they could be happier and friendlier.

“If you’re constantly hiding things or whatever, you know, your negativity kind of comes in because you know, you can get more bitchy or whatever. But I mean like here, it’s like you know, if you’re positive then you know positive emotions come out and stuff so you’re happier and you’re more friendly.”" – Participant #12

Dan described the changes he had seen in himself and others and noted that he was starting to care again:

“You get tired of asking, you get tired of giving it, you get tired of caring. I started to care again...[others are] pretty much the same as me. They started to trust again. They started to care. Both about other people and of themselves.” - Dan

He credited the staff for this shift in perspective and represented this as light shining through the darkness.
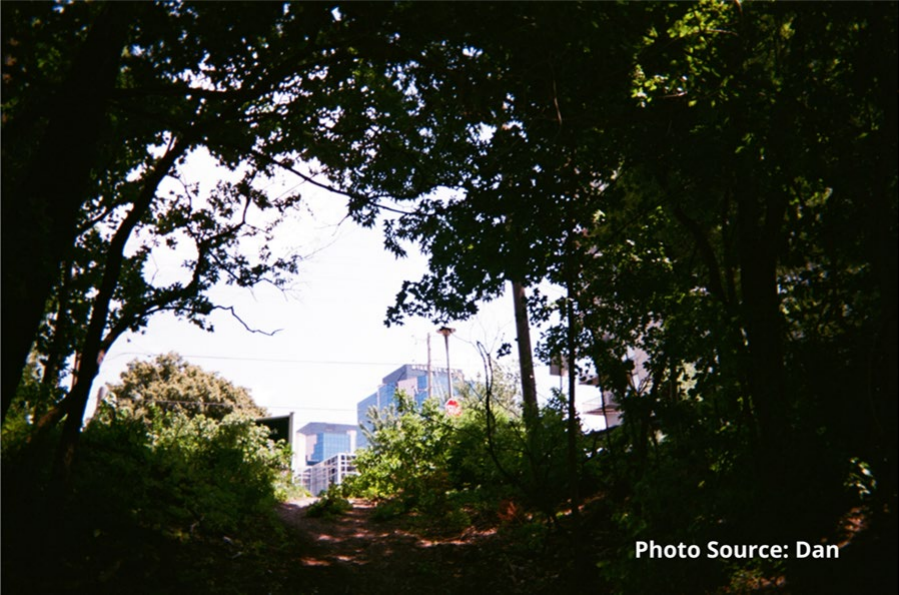

“They put faith in me when no one else would” - Dan.

Participants found that the site was unexpectedly impacting their day-to-day well-being and how they take care of themselves. The staff’s acceptance of participants appears to be at the root of these changes to well-being. Participants now see the site as a place to go to lift their mood and shift their perspective.

“Well coming to this place got me introduced to health and the first people I met here was able to find housing for me.... It meant a lot that someone actually cared for my wellbeing. It was good. It was a good feeling that there’s people like that here that will help people.” – Participant #5

Personal transformations also influenced how people now participated in community:

“I’ve noticed more love and respect to the point it’s becoming a little family.... They take care of each other. They look out for each other.” - James.

Several participants noted that due to improvements in their lives as a result of the site, they could now give back to the community in a variety of ways. Some encouraged others within their peer group to use substances more responsibly and to use at the site, rather than in public. In this way, the work of the site extended into the community; those who had used the site became advocates for safer practices, thereby reducing negative impacts of substance use.

One of the ways participants were looking out for each other was to reverse overdoses. With the increased number of overdoses, many participants had saved the lives of their friends using naloxone. Although this can be a complex dynamic, participants reported that they felt better knowing they had saved someone’s life. As the participants journeyed from survival, to belonging, to starting to care more about themselves and others, they also started to care about how they are perceived in the community. Some participants had become engaged in cleaning up discarded gear in the community both through formal volunteering or more informally:

“Yea, there’s peoples right out, if I’m walking around or whatever, if I see a dirty on the street I always pick it up, I always see random dirty rigs, but I’d rather pick it up and bring it here you know.... Or if I’m at people’s houses and they have dirty bins that are full, I always offer to take them down here.” - Legend.

For one participant, taking part in the needle recovery program run by the site gave him opportunities to learn responsibility and give back to society:
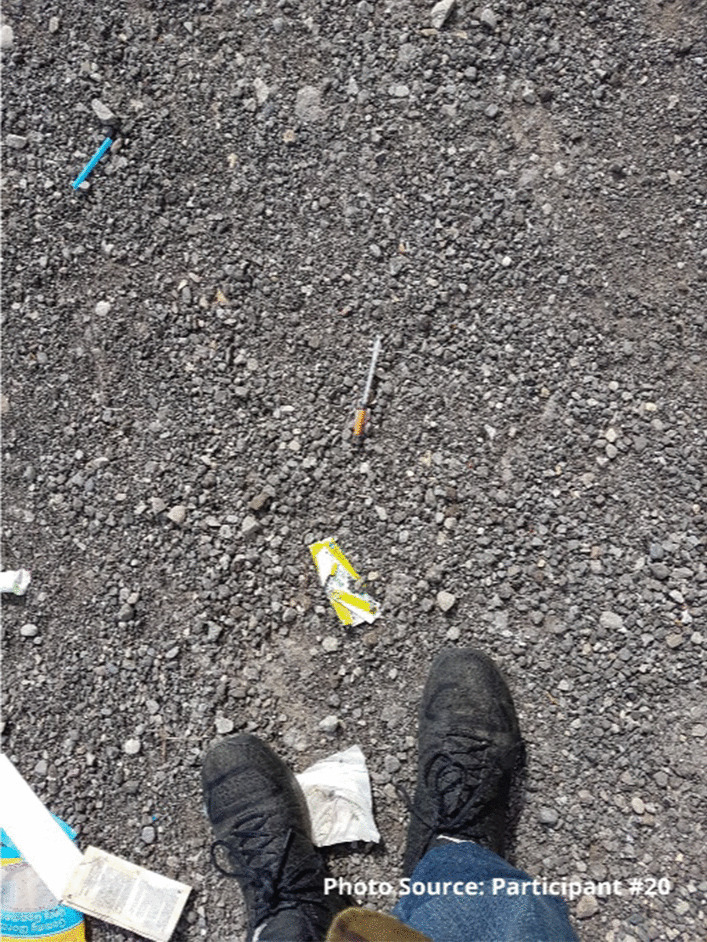

“That showed me responsibility when I had to go to pick up dirty needles. It’s a responsibility to show me some work ethic too.... All my life it depends on doing drugs, doing drugs, doing drugs. At least I got to go out with some people and get to talk, we got to work and do something positive and do something that’s going to help the community because my goal, if I ever do become sober, I want to give back to the place I came from, to the people that helped me.” Participant #20

Participants spoke about the fact that substance use is still a highly stigmatized experience. They described negative encounters with both the general public and with health professionals. Many felt the stigma in society daily, but saw the site starting to change the story about substance use. They wanted the public to know how important the site was to their journey.

“They [the public] need to know what this place is offering people and how it’s changing people’s lives. It’s not promoting use. It’s providing a safe place if you choose to do so, and they have all the avenues to help you get out of your slump, and they have the connections to get you into treatment, they encourage you, if you choose to do so.” - Participant #27.

For some participants, their experiences at the site provided opportunities to find hope or new directions. One participant addressed the positive energy derived from the site and noted its health-promoting influence:
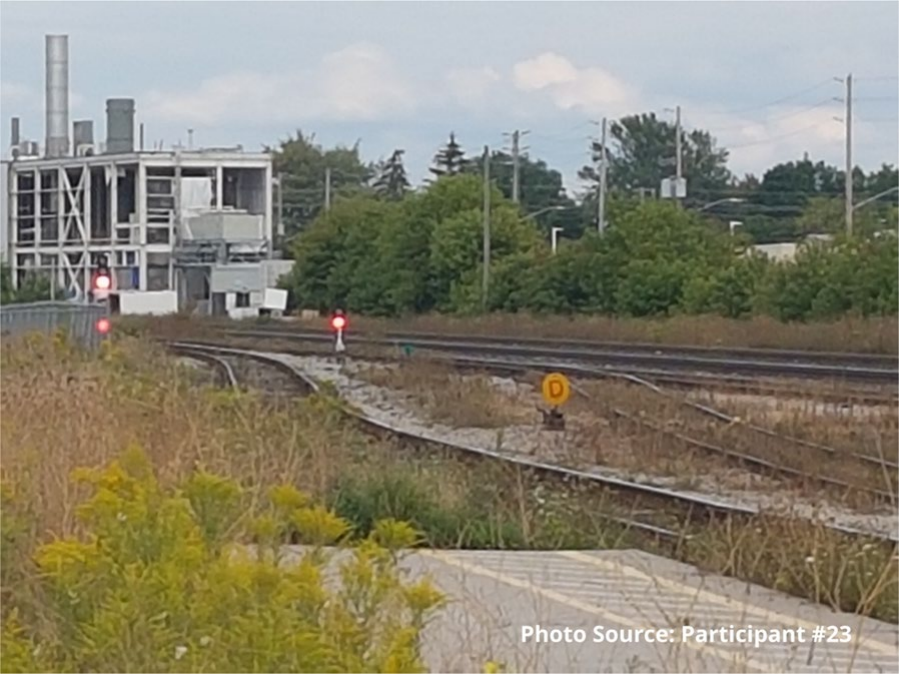

“The site is what we’re talking about, it’s about what saved my life basically... They help me want to go in the right direction. That’s why the train tracks are there. So recently, and most of the time I’m here, it’s like positive energy you know. It makes you want to be better and go in the right direction, and just recently I chose, I want to go to rehab.” - Participant #23

Another participant wanted to highlight how the site provides the space that can inspire individuals to grow. For this participant, coming to the site gives her hope for a positive future:
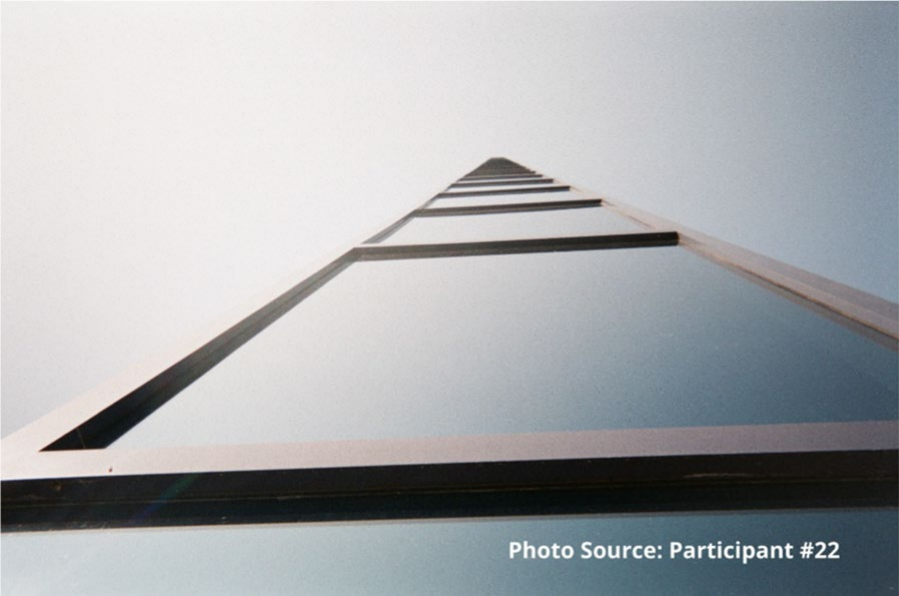

“The sky’s the limit right, I can do anything... that’s the hope I feel when I walk into these doors. Every time I walk in this door, this place saves my life, every time. Even if I didn’t even realize I wasn’t having a good day, this place will make me feel better. Every time.” – Participant #22.

These stories express opportunity and hope. The atmosphere of safety, connection, and belonging provided at the site led participants to unexpected transformations.

## Conclusions

While participants provided in-depth insight into the meaning of the site in their lives, there were some limitations faced in the project. Firstly, we initially had low follow-through from participants in taking and returning photographs. This was mitigated by including a peer support worker to assist with taking the pictures and returning the cameras. Secondly, much of the discussion related to relationships with site staff but our only participants were site users. A more developed perspective of relational practices could be developed by including interviews with staff and management. Finally, our analysis was concluded through the outset of the Covid-19 pandemic. While this did not impact data collection, the site context may have conceivably changed over the past several months of the pandemic and might impact upon future policy and program directions.

Accessing services at an overdose prevention site allowed our study participants to derive a degree of safety. Our research found that the site enhanced participants’ physical safety through access to health professionals and harm reduction practices, such as clean facilities, new injection equipment, and access to naloxone. Additionally, our research found that physical safety was improved through an “unrushed” injection process free from the fear of being arrested or assaulted. Congruent with Kerman et al. [17], in addition to the physical safety offered at the site, participants clearly conveyed “feeling safe” at the site. Participants poignantly told us that the site offered a stigma-free environment of acceptance, connection, and belonging. One of the service provision partners at the site was an indigenous health and wellness organization. Some participants spoke to how getting connected with Indigenous Wellness Workers was helping them connect to cultural and land as part of their feeling safe and ultimately transforming. The care participants received at the site was in direct contrast to their experiences of stigmatization, marginalization, violence, and unpredictability that they endured elsewhere in the community. We use the term “psychological” safety, the ability to show and employ one’s whole self without fear of negative consequences [10], to capture the full essence of what our participants were saying. Although the term psychological safety has been primarily used in organizational behaviour research, it is applicable to how participants experienced a caring and trusting environment and felt included at the site without the fear of being marginalized. Similarly, previously conducted research has reported that these sites offer a safe, non-judgemental, and supportive environment [14, 19, 22, 24] providing site users with a sense of belonging and connection with others [8]. The current findings also align with earlier studies that found these facilities can mediate access to other services and supports including, food, shelter, and additional health and social supports [14, 19, 22].

Our research contributes to an enhanced understanding of how caring relationships with staff at the overdose prevention site impacted the site users’ sense of self. Caring relationships as an intervention have a long history in health professions [23, 31]. What we propose is that caring relationships are an intervention in and of themselves and that these relationships contribute to transformation that extends far beyond the public health outcomes of disease reduction. The caring relationships at the site begin to address existing social barriers to achieving health and well-being experienced by the site users. For indigenous participants, this included caring relationships with cultural health workers. For all participants, these relationships are transformative in unexpected ways and can be the starting point for significant life changes, such as secured housing, employment and controlled or discontinued substance use. Prior to using the site, many participants felt that no one cared for or about them, and they similarly did not care for themselves. Participants knowingly engaged in risky substance use practices without concern for the consequences. However, when participants perceived the staff to be accepting and compassionate towards them, they started to feel valued. This acceptance and compassion were demonstrated by staff through the provision of care that was free of stigma, discrimination, blaming, or shaming. Participants started to feel like “*normal human beings*” and their perception of self-improved. Over time, participants began to take steps towards a healthier future, a process that is often one of incremental gains and on individualized continuums. Our findings show that caring relationships between staff and site users and the way care was provided, meant site users were considering healthier choices and caring about the consequences of their choices. By simply knowing that someone cared for them, participants started to make changes including using substances in a safer manner or reducing their substance use. Caring relationships between staff and site users were the intervention that triggered these initial steps towards personal transformation.

The site played an important role in bringing stability to participants’ lives, such sites are more than just disease prevention facilities [17]. The site has become a refuge and safe-haven from the daily chaos participants face in their lives. For many participants, the site became part of their daily routine, especially for those site users experiencing homelessness. The consistent care and compassion that the participants received at the site, brought calm and stability to their lives, even if it was just for a moment. The stability that the site offered created opportunity for healthier behaviours. Congruent with Kimber et al. [18], participants gave examples of controlling when and how much substance they used, starting to follow regular eating and sleeping patterns, finding housing, or beginning drug replacement therapy. Participants were exercising more control over their lives, and some described their hopes and dreams for the future. The regular connection with the site, combined with the caring relationships found there, supported a shift away from chaos. The stories and photographs shared by participants show how some participants have started to think about their future from a place of stability.

As participants started caring for themselves, that caring has extended to the community in which they live. Our findings suggest that harm reducing practices extended well beyond the doors of the site. Bennett et al. [2] note that as many as 23% of peers who are provided with a naloxone overdose prevention kit use this kit and return for a replacement. Similarly, in our study many participants have routinely reversed overdoses experienced by their peers. While this can be a traumatic experience, many also felt that this was a way for them to give back to their community. Participants have also taken on monitoring roles to encourage their peers to dispose of used gear in designated bins and to use the site rather than injecting in public. Others expressed the desire to “give back to the site” by volunteering their time in the needle recovery program. As participants started caring for themselves, that caring has extended to the community in which they live. The site, like others [17], appears to have facilitated the community coming together in unprecedented ways placing overdose prevention sites in a unique position to engage people who use substances as allies in health promotion and become a jumping off point for civic engagement. As experiences in Vancouver’s substance use community have shown, people who use substances have a desire to be meaningfully involved in the response to the drug crisis [34].

These findings highlight the transformational impact that overdose prevention sites can have on the lives of site users. Caring relationships with staff served to increase stability in the lives of site users and enhanced their relationships with their peers and the community. However, when site users re-enter the community, they are again confronted with oppressive systemic issues, within health and social services and beyond, which continue to place blame and shame on them and create barriers to achieving the determinants of health.

Similar to other samples of PWUD [15], our participants described multiple life challenges such as homelessness, unresolved physical and mental health issues, and lifelong trauma. Many alluded to complex events that led them to the path of substance use for survival. Their stories about the lives they endured contained experiences of violence, mental illness, discrimination, and marginalization. Their experiences reflected the systemic and interpersonal oppression they experienced, often in multiple ways, because of their substance use and other overlapping social identities including race, gender, housing status, and disability. It is important to explore how intersecting social identities influence health outcomes and enhance health disparities. Previous research has noted that these intersecting social identities play an influential role in perpetuating health disparities among disadvantaged groups of people [5]. Stigma resulting from addiction to drugs can be further exasperated when it intersects with other forms of bias such as racism and sexism. Therefore, we recognize importance of social identities in shaping the day-to-day experiences of our participants while accessing the site. We also realize that individuals may encounter even more adverse circumstances and experiences based on their social identifiers. Future analyses of our data will examine in more detail how various social locations and identities shaped the experiences of site users and their ability to access needed services.

Through this work, we want to build a shared understanding of a complex problem. While the site was set up as a public health intervention to reduce harms caused by substance use, it has functioned as much more than this. In addition to the physical safety the site provided, the site helped participants feel safe because of the way service is delivered. Through caring relationships, the overdose prevention site provided a sense of connection and belonging for participants. The caring relationships developed with staff helped participants feel valued. It is from this place of being valued that participants encountered what we have labelled as “unexpected transformations”. For many non-substance users, it can be easy to blame substance users for their addiction. We hope this arts-based approach to sharing the context of our participants’ daily lives can be a tool for fostering reflection and conversation in the community, a discussion that brings the people who use substances into the conversation. Through learning about their experiences, we hope that our community can see the elements that are working well in the site, including the caring relationships, the increasing stability, and the community engagement, and collectively build new and meaningful ways to create a better future for all involved.

## Data Availability

The data sets generated and/or analysed during the current study are not publicly available due to privacy and confidentiality related to qualitative and photograph data.

## Abbreviations

PWUD: People who use drugs
