# Correction to: A critical narrative inquiry to understand the impacts of an overdose prevention site on the lives of site users

**DOI:** 10.1186/s12954-021-00464-w

**Published:** 2021-02-08

**Authors:** Abe Oudshoorn, Michelle Sangster Bouck, Melissa McCann, Shamiram Zendo, Helene Berman, Jordan Banninga, Marlene Janzen Le Ber, Zayya Zendo

**Affiliations:** 1 Western University, London, Canada; 2 Middlesex-London Health Unit, London, Canada; 3 Centre for Research on Health Equity and Social Inclusion, London, Canada; 4 School of Leadership and Social Change, Brescia University College, London, Canada

## Correction to: Harm Reduct J (2021) 18:6 10.1186/s12954-020-00458-0

After publication of the original article [1], the authors identified an error in the author name of Marlene Janzen Le Ber. The given name and family name were erroneously transposed.

The incorrect author name is:

<GivenName>Marlene J.<GivenName>

<FamilyName>Le Ber</FamilyName>

The correct author name is:

<GivenName>Marlene Janzen</GivenName>

<FamilyName>Le Ber</FamilyName>

The original article has been corrected.
